# Vibration Control of Cylindrical Piezoelectric Transducers Utilizing Stepped-Thickness Configurations

**DOI:** 10.3390/s26165240

**Published:** 2026-08-19

**Authors:** Ata Meshkinzar, Ahmed M. Al-Jumaily

**Affiliations:** 1 Department of Mechanical Engineering, Auckland University of Technology, Auckland 1010, New Zealand; ahmed.aljumaily@aut.ac.nz; 2 AUT Institute of Biomedical Technologies, Auckland University of Technology, Auckland 1010, New Zealand

**Keywords:** sensing, actuation, piezoelectric transducers, cylindrical transducers, stepped-thickness transducers, biomedical transducers, food industry transducers

## Abstract

Piezoelectric transducers have been extensively investigated for their widespread use in a variety of sensing and actuation applications. Among them, cylindrical piezoelectric transducers have received less attention despite their strong potential. The aim of this work is to investigate vibration mode shape control by introducing axial and circumferential steps in the thickness of these transducers. The efficacy of introducing these steps has been investigated through ANSYS simulations and is subsequently validated using Laser Scanning Vibrometer to obtain the mode shapes experimentally. Electrical impedance measurements have also been done to obtain the resonance and anti-resonance frequencies and the effective electromechanical coupling factor. The results show that steps can control vibration through two mechanisms. (i) Circumferential steps can excite modes whose circumferential mode numbers match the number of steps. These specific modes occur within a frequency range that is entirely absent in uniform thickness transducers. (ii) Axial steps can localize and amplify the vibration amplitude for axial vibration modes. Having validated the efficacy of these steps in controlling vibration modes, some experiments were done to evaluate the effect of these steps on the performance of these transducers as acoustic actuators. The strength of the acoustic field generated inside these transducers was measured experimentally using a precision microphone and it was shown that the stepped transducers can generate around 1.5 times stronger acoustic field as compared to the uniform thickness transducer. Electrical impedance results revealed that stepped transducers had lower effective electromechanical coupling factors. However, this may suggest they can provide narrowband transducers which may have numerous precision applications. These results may suggest that employing steps could control vibration, enhance the performance of cylindrical piezoelectric transducers and improve their uptake as sensors or actuators for biomedical or food industries as well as other industrial applications.

## 1. Introduction

With numerous applications in biomedical and food sectors, non-destructive-testing (NDT) and many more industrial fields, piezoelectric transducers come in various geometries and configurations as well as materials to suit this wide range of applications. Although there has been much research on material development for various transducers which is beyond the scope of the present work, there has also been continuous research on improving transducer performance by enhancing its sensitivity for sensing applications or strengthening its actuation capabilities such as its generated acoustic field [1]. To this aim, various geometries and configurations at different micro/macro scales have been investigated including rectangular and circular plates, arrays of transducers, and curved and stepped plates [2,3,4,5,6,7,8,9]. For instance, to improve the focus of the radiated waves, curved plates were employed; however, they had two vibration modes, namely in-plane and flexural, which coupled, leading to a restriction in their uptake [5,10,11]. Modifying the geometrical parameters of the transducer could help avoid this mode coupling but may not be possible due to other design considerations and restrictions. Employing a circular array of transducers is another approach; nonetheless, even small variations in dimensions alter the focal line of the transducer [7]. There are several studies focusing on step-wise thickness variations in flat circular and rectangular plates which result in counter-phase radiations becoming in-phase, getting rid of phase cancelations [2,3,6,12,13,14]. The regions which were initially vibrating in counter-phase for the uniform thickness plate had thickness variations in a half wavelength so that the vibrations and radiations became in-phase.

Another approach is a cylindrical shell transducer which has been investigated in the literature for various purposes and using different materials. An example is a metallic cylindrical shell driven by a shaker to dry fruits by acoustically removing the moisture content but had expensive and bulky power and electric drive requirements [8]. Piezoelectric cylindrical shells have been investigated in various studies without the need for such expensive and bulky power drive requirements [1]. For instance, Ostasevicius et al. employed a piezoelectric cylindrical transducer to acoustically separate microparticles from a suspension [15]. Cylindrical geometry enhanced the focus of the radiated waves. However, to further improve the performance of the transducer for generating focused acoustic field, there have been other attempts and investigations in the literature. Stepped-thickness flat plates, as stated above, have shown improvements in the acoustic radiation performance. Stepped-thickness cylindrical shells with various types of axial or circumferential steps were also investigated in the literature. Some of these studies focused only on theoretical analysis and different mathematical techniques for the free and forced vibration of these stepped-thickness shells [16,17,18,19,20]. However, some others focused on the acoustic radiation characteristics of such transducers both experimentally and using ANSYS simulations and concluded that stepped-thickness cylindrical transducers can enhance the acoustic field similar to what was reported for stepped plates once designed properly [21,22,23,24]. An application of such a stepped cylindrical transducer was to acoustically enhance the evaporation of polydisperse micro water droplets generated by a first-stage nebulizer enabling their use in the biomedical sector, for airway humidification or drug delivery [25]. In another investigation, the potential of such transducers was investigated for acoustic emulsion destabilization in the dairy sector to separate fat from milk [26].

In spite of the above research works, there is very limited research available in the open literature on the vibration of stepped-thickness piezoelectric cylindrical transducers [24]. Since the uptake of such transducers is on the rise and there is strong potential for more widespread use of them in various sectors and for different applications [1], there is a strong need to better understand their vibration characteristics, the effect of various axial and circumferential steps on the vibration mode shapes, amplitude and generally vibration control of these piezoelectric cylindrical transducers. The physical phenomenon that the authors are trying to investigate is whether these axial and circumferential steps can practically localize vibration amplitude within the stepped regions and excite certain mode shapes which were absent in the uniform thickness transducer within the same frequency range of operation. To this aim, a comprehensive investigation is performed on piezoelectric cylindrical transducers with various numbers of axial and circumferential steps using both simulations in ANSYS as well as experiments for validation. The aim is to better elucidate their vibrational behavior which could be of strong importance for their performance in a variety of applications, some of which were elaborated on earlier.

## 2. Materials and Methods

### 2.1. Materials

A PZT-5L circular cylindrical shell manufactured by Nanjing Hanzhou Technologies Co., Ltd. (Nanjing, China) with dimensions of 30 mm outside diameter, 26 mm inside diameter and 50 mm length was purchased and subsequently machined to create the required step-thickness configurations. The machined samples were recoated with a very thin layer of silver epoxy as the electrode. Table 1 presents the material properties [27].

### 2.2. Methodology

**Figure 1:**
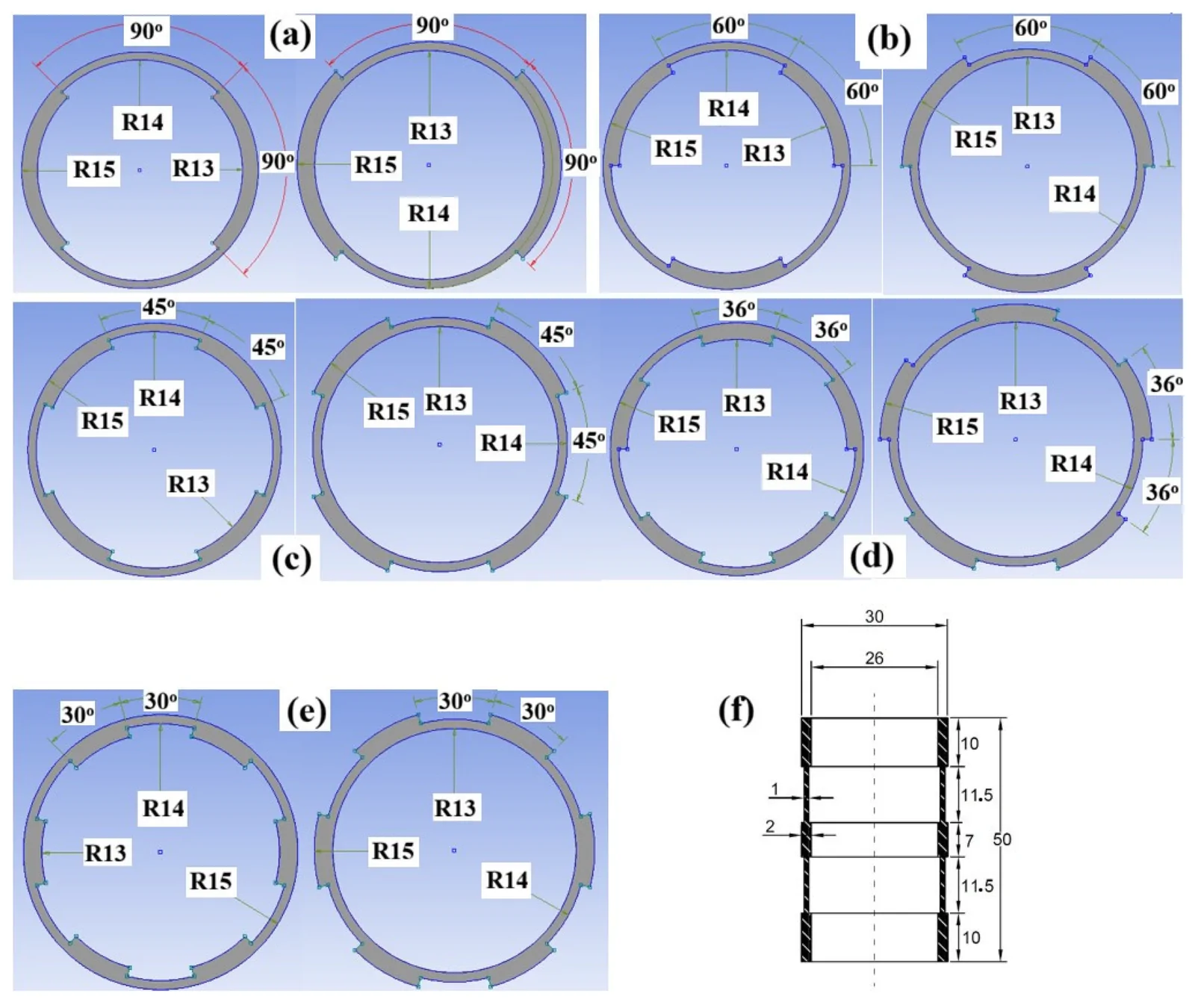
Schematic of stepped-thickness piezoelectric transducer configuration with (**a**) two internal/external circumferential steps, (**b**) three internal/external circumferential steps, (**c**) four internal/external circumferential steps, (**d**) five internal/external circumferential steps, (**e**) six internal/external circumferential steps and (**f**) two internal–external axial steps (all dimensions in mm).

In this work, both axial and circumferential steps are introduced to investigate their effect on the vibration mode shape control. For the circumferential case, transducers with two, three, four, five and six steps on the inner and outer surfaces are considered. For each of these, the stepped and non-stepped regions are equally spaced around the circumference as in Figure 1. The aim is to investigate whether certain mode shapes with circumferential wave numbers equal to the number of steps would be forcibly excited as a means to vibration control. For the axial case, a transducer with two steps on the inner and outer surfaces is considered. To clarify how the dimensions and step locations are identified for the case of axial step in Figure 1, it is first required to perform an analysis in ANSYS on the uniform thickness transducer to obtain the mode shape with axial mode number of 5 where there exist regions which are out-of-phase. By creating steps within those regions, the aim is to investigate the effect of these steps on the vibration mode shape and amplitude localization.

### 2.3. Simulation Details

ANSYS software 2025 R2 was employed to investigate the vibration characteristics of the transducers and simulations were performed using harmonic module. Since the boundary conditions affect the vibration characteristics, some iterations of simulations were performed to identify the most suitable boundary condition which best resembles the experimental setup used and satisfies the experimentally observed result for the breathing mode of the uniform thickness transducer at around 38 kHz. Accordingly, simply supported boundary conditions were identified as the most accurate and were used in simulations. A measurement of 36 V of excitation voltage was applied to the outer surface of the piezoelectric transducer while the inner surface was grounded (zero voltage applied). A minor damping of 0.01% was introduced to ensure numerical stability and convergence at resonant frequencies. The aim of the study is not to quantify the damping or the absolute deformation amplitudes but rather to investigate the vibration mode shape control and localization by introducing steps. Hence, the vibration amplitudes should be treated as relative values and not absolute. Normalized amplitude graphs are also provided for the sake of relative comparison. Frequency was swept from around 30–50 kHz to identify the potential localized mode shapes. Meshing is also an important consideration particularly with the steps introduced in the thickness. As per ANSYS standards [28], mesh skewness of less than 0.95 is acceptable with lower values being of higher quality. All the element and mesh details for all transducers are presented in Table 2.

It is also worth considering the theory behind piezoelectric materials and how ANSYS handles them. Constitutive relations for linear piezoelectric materials can be stated in the matrix form as [29]

(1) $$\left\{T\right\}=\left[c^{E}\right]\left\{S\right\}-\left[e\right]\left\{E\right\}$$

(2) $$\left\{D\right\}=\left[\varepsilon^{S}\right]\left\{E\right\}+{\left[e\right]}^{T}\left\{S\right\}$$

where { } denotes vector and [ ] denotes a matrix, superscript T is transposed matrix or vector, $\left\{T\right\}$ is the stress vector, $\left\{S\right\}$ is the strain vector,$\left[c^{E}\right]$ is the stiffness matrix at constant electric field, $\left[e\right]$ is the piezoelectric stress constant matrix, $\left\{E\right\}$ is the electric field vector, $\left\{D\right\}$ is the electric displacement vector and $\left[\varepsilon^{S}\right]$ is the dielectric permittivity matrix at constant strain.

The structural dynamic behavior of the transducer is governed by Newton’s equation of motion:

(3) $$\nabla .\left\{T\right\}+\left\{f\right\}=\rho \ddot{u}$$

where $\left\{f\right\}$ is the body force vector, $\left\{u\right\}$ is the 3D displacement vector and ρ is the material density.

The electrical behavior satisfies Gauss’s law for electrostatics:

(4) $$\nabla .\left\{D\right\}=0$$

and the electric field $\left\{E\right\}$ is related to the electrical potential Φ by

(5) $$\left\{E\right\}=-\nabla \Phi$$

For harmonic analysis in frequency domain, time-dependent structural displacement and electrical potential can be written in the complex form as $\left\{u(t)\right\}=\{U\}e^{i\omega t}$ and $\left\{\Phi (t)\right\}=\{ɸ\}e^{i\omega t}$ where {U} and {ɸ} are the corresponding time-independent amplitude vectors and ω is the frequency of vibration.

In ANSYS, once the finite element discretization is done, the following dynamic system of equations in the matrix form is obtained:

(6) $$\left[\begin{array}{c} {}[M]\;\left[0\right]\\ \left[0\right]\;\;\;[0] \end{array}\right]\left\{\ddot{\begin{array}{c} \left\{u\right\}\\ \ddot{\left\{\Phi \right\}} \end{array}}\right\}+\left[\begin{array}{c} {}[C]\;\;\;\left[0\right]\\ \left[0\right]\;\;\;[0] \end{array}\right]\left\{\dot{\begin{array}{c} \left\{u\right\}\\ \dot{\left\{\Phi \right\}} \end{array}}\right\}+\left[\begin{array}{c} {}[K]\;\;\;\;\;\;\;\left[K^{z}\right]\\ {\left[K^{z}\right]}^{T}\;\;\;[K^{d}] \end{array}\right]\left\{\begin{array}{c} \left\{u\right\}\\ \left\{\Phi \right\} \end{array}\right\}=\left\{\begin{array}{c} \left\{F(t)\right\}\\ \left\{Q(t)\right\} \end{array}\right\}$$

where [M] is the mass matrix, [C] is the structural damping matrix, [K] is the structural stiffness matrix, $\left[K^{z}\right]$ is the piezoelectric coupling matrix (derived from the stress matrix $\left[e\right]$), and $\left[K^{d}\right]$ is the dielectric permittivity matrix (derived from $\left[\varepsilon^{S}\right]$), $\left\{F(t)\right\}=\{F\}e^{i\omega t}$ is the mechanical force vector and $\left\{Q\left(t\right)\right\}=\{Q\}e^{i\omega t}$ the electrical charge vector.

Upon substituting $\left\{u(t)\right\}$, $\left\{\Phi (t)\right\}$ and their derivatives into (Equation (6)), one can obtain the final frequency-domain equation that ANSYS solves at every frequency step as

(7) $$\left[\begin{array}{c} \left[K\right]-\omega^{2}\left[M\right]+i\omega [C]\;\;\;\;\;\;\;\left[K^{z}\right]\\ {\left[K^{z}\right]}^{T}\;\;\;\;\;\;\;\;\;\;\;\;\;\;\;\;\;\;\;\;\;\;\;\;\;\;\;\;\;\;\;\;\;\;\;\;\;[K^{d}] \end{array}\right]\left\{\begin{array}{c} \left\{U\right\}\\ \{ɸ\}\; \end{array}\right\}=\left\{\begin{array}{c} \left\{F\right\}\\ \left\{Q\right\} \end{array}\right\}$$

### 2.4. Experimental Details

**Figure 2:**
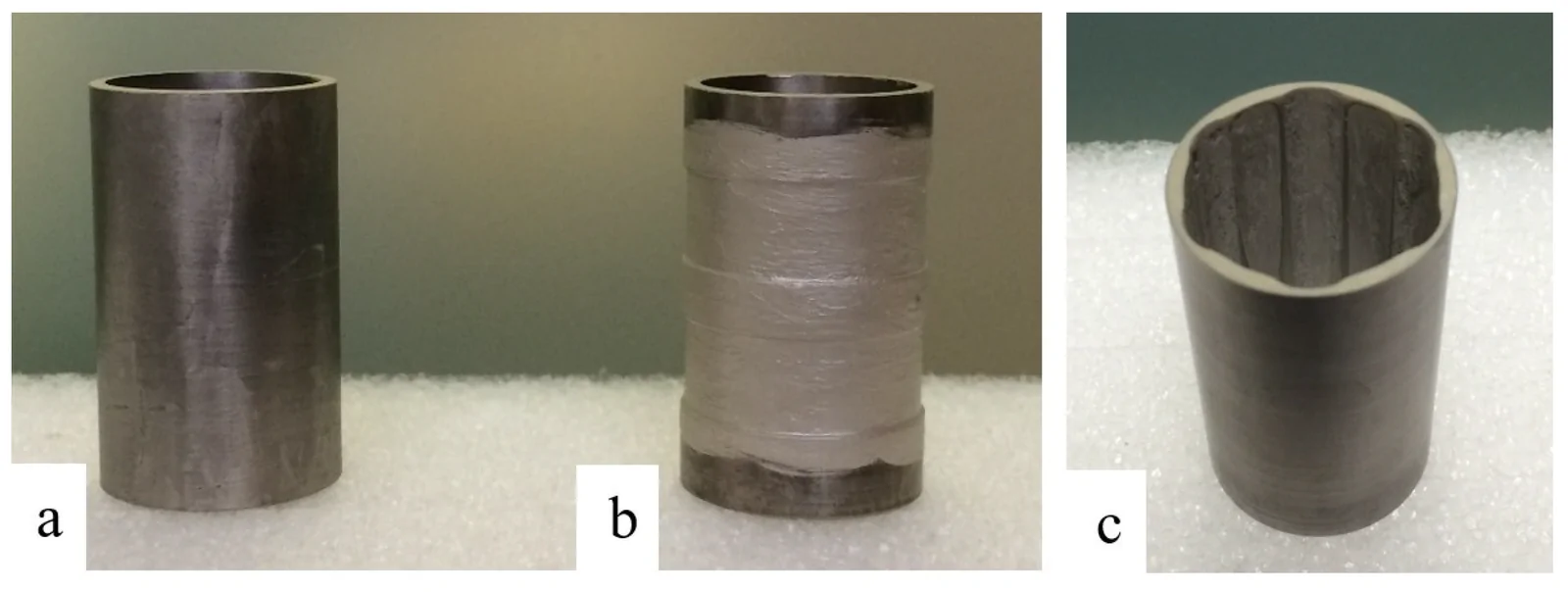
Experimentally tested transducers: (**a**) Uniform thickness; (**b**) two internal–external axial steps; (**c**) six internal circumferential steps.

**Figure 3:**
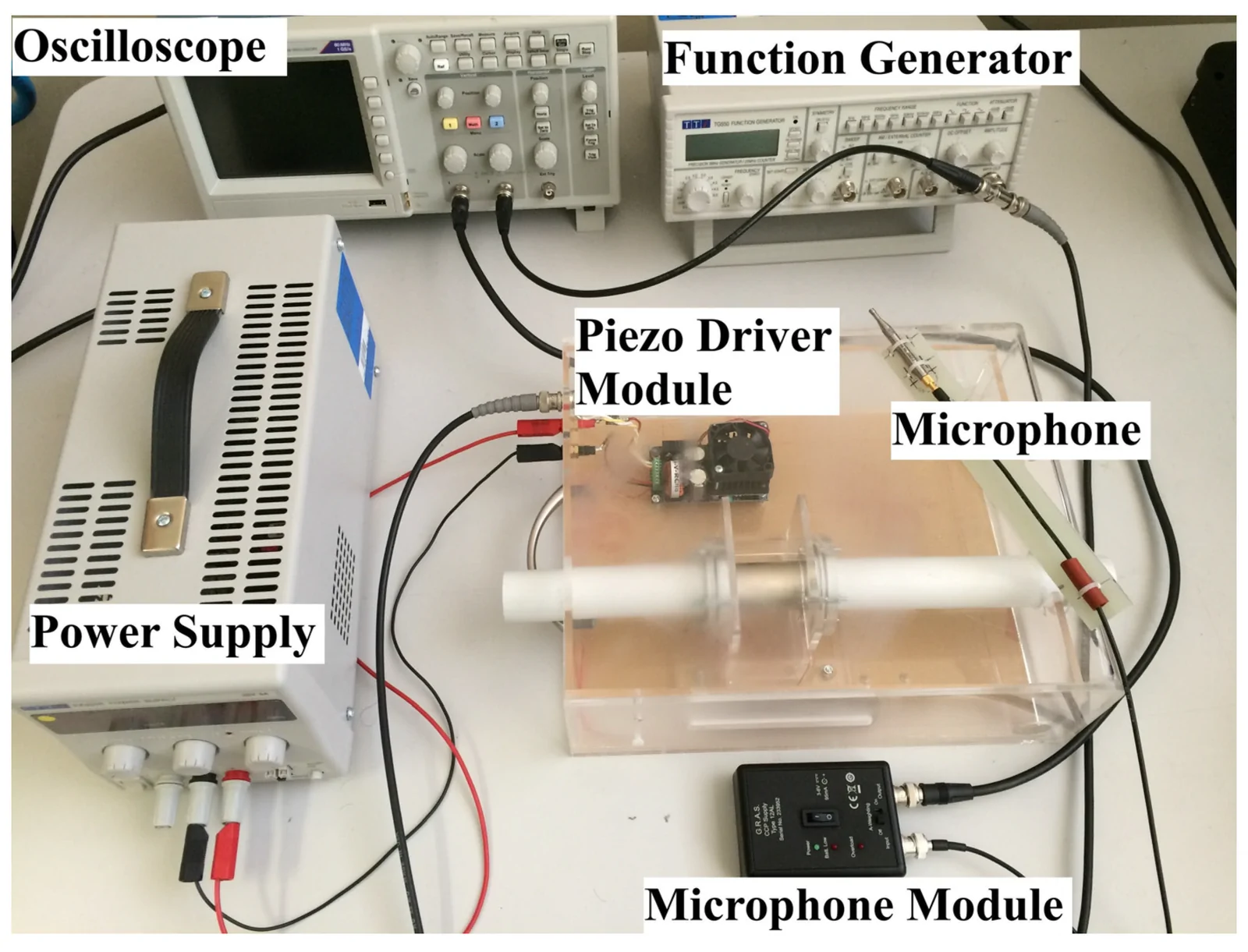
Experimental setup.

A small diameter diamond-grinding wheel, operating at approximately 15,000 rpm, was used for machining the transducers and the machined regions were subsequently recoated with a thin layer of silver epoxy as the electrode. These piezoelectric transducers are extremely crack-prone; hence, machining them is very cumbersome. Accordingly, other than the uniform thickness transducer, only one with axial and one with circumferential steps were experimentally tested which are illustrated in Figure 2. Transducers were driven using a piezo driver module supplied with a sinusoidal waveform of different frequencies within the range of 30–50 kHz by a function generator providing 3.6 V_p-p_ voltage. The piezo driver module has a gain of 20 so 72 V_p-p_ is delivered to the transducer similar to the 36 V peak defined in ANSYS. The piezo driver itself is driven by a DC power supply at 24 V. The experimental setup is shown in Figure 3. As seen, the whole transducer setup is enclosed in a transparent acrylic box for safety purposes. A G.R.A.S. 46DD 1/8″ CCP Pressure Standard Microphone Set (Holte, Denmark) with a 12AL G.R.A.S. CCP Power supply module is also employed to evaluate the performance of the transducers as an acoustic actuator by measuring the acoustic field generated inside the transducers. White guideline pipes are also considered as a safe path to insert the microphone into the transducer for acoustic measurement. To avoid interference of the microphone with the acoustic field inside the transducer, the setup has been designed in a way that only the narrow tip of the microphone gets in the transducer.

To assess the electrical performance of the transducers, an impedance analyzer was used to measure the frequency response and identify resonance and anti-resonance frequencies.

To capture the operating deflection shapes (ODS) of the transducer, a Polytec PSV-400 Scanning Laser Doppler Vibrometer (Waldbronn, Germany) was employed. The system was configured to operate in its native, single-frequency ‘FastScan’ mode while the transducer was continuously driven by the piezo driver stated above. The laser beam scanned sequentially across a user-defined geometric mesh mapped directly over the transducer’s surface. At each discrete measurement node, data acquisition was automatically phase-locked to the excitation source frequency via the software’s internal least-squares regression architecture, completely eliminating spectral leakage while maintaining absolute spatial phase coherence across the entire scanned geometry.

## 3. Results and Discussion

First, the uniform thickness transducer was analyzed in ANSYS to identify its vibration mode shapes, and in particular to identify whether circumferential mode shapes occur within the low-ultrasound frequency range of 30–50 kHz for that particular size transducer. Subsequently, circumferentially stepped transducers with different numbers of internal or external steps were investigated. Further, an axially stepped transducer with two internal–external steps was investigated to evaluate the effect of introducing the steps on the vibration mode shape. The following sections elaborate on the obtained results.

### 3.1. Simulation Results

**Figure 4:**
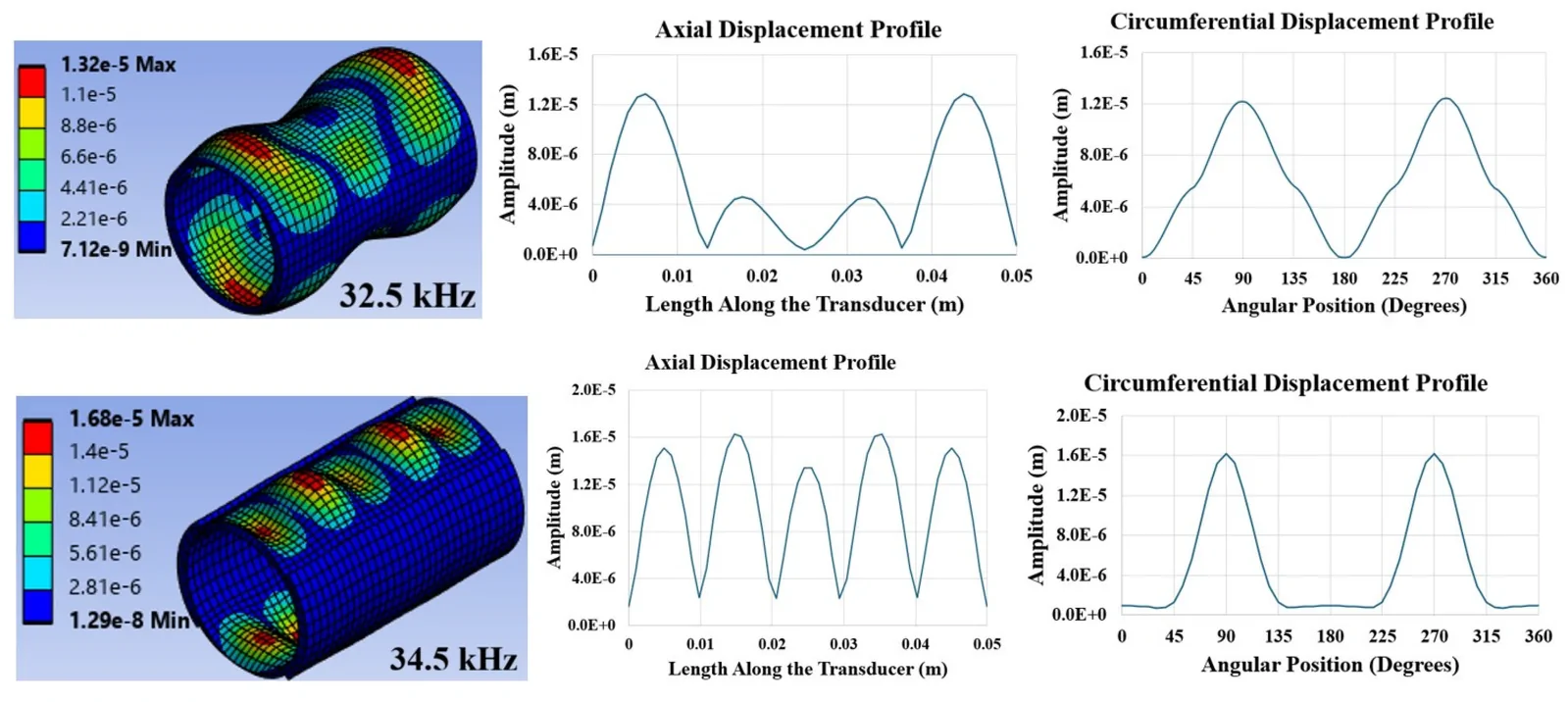
Mode shapes in 3D (deformations in meter), axial and circumferential wave profiles. (**Top**): Two internal circumferential steps at 32.5 kHz. (**Bottom**): Two external circumferential steps at 34.5 kHz.

**Figure 5:**
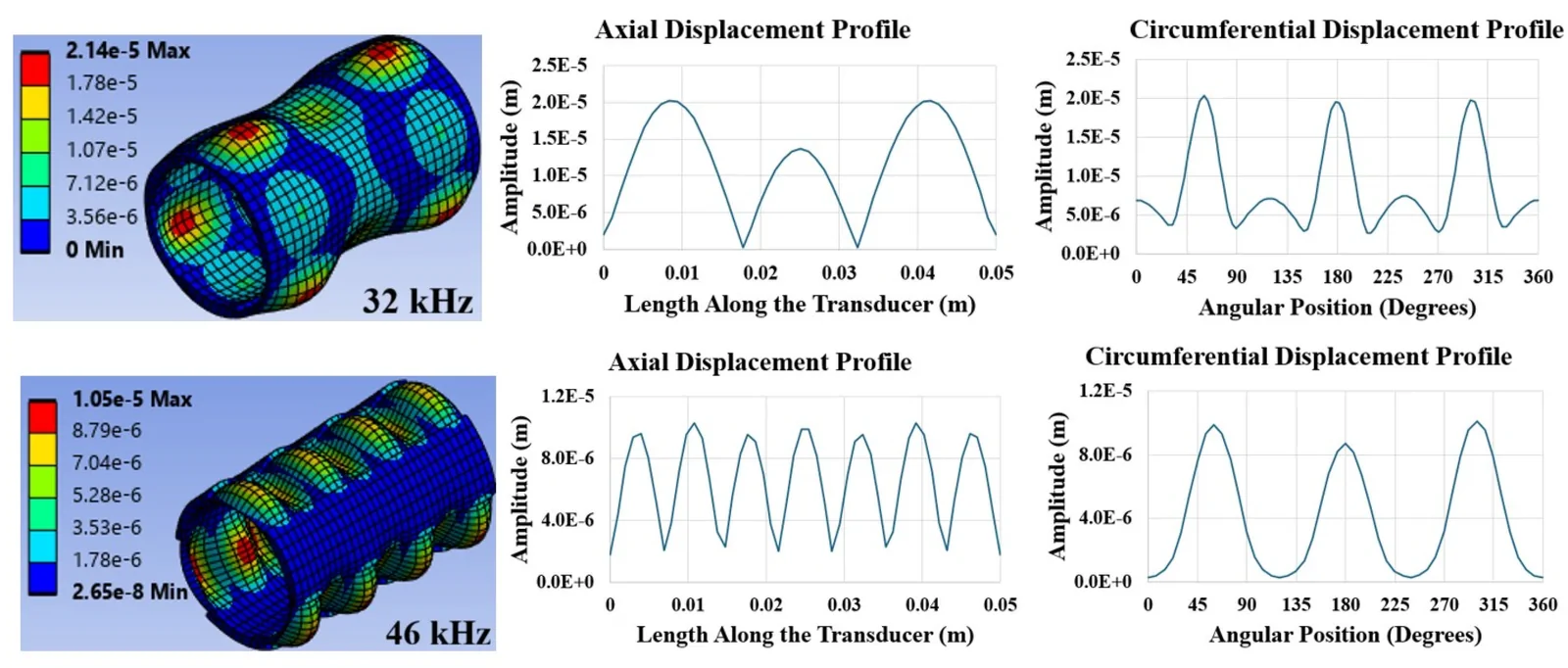
Mode shapes in 3D (deformations in meter), axial and circumferential wave profiles. (**Top**): Three internal circumferential steps at 32 kHz. (**Bottom**): Three external circumferential steps at 46 kHz.

**Figure 6:**
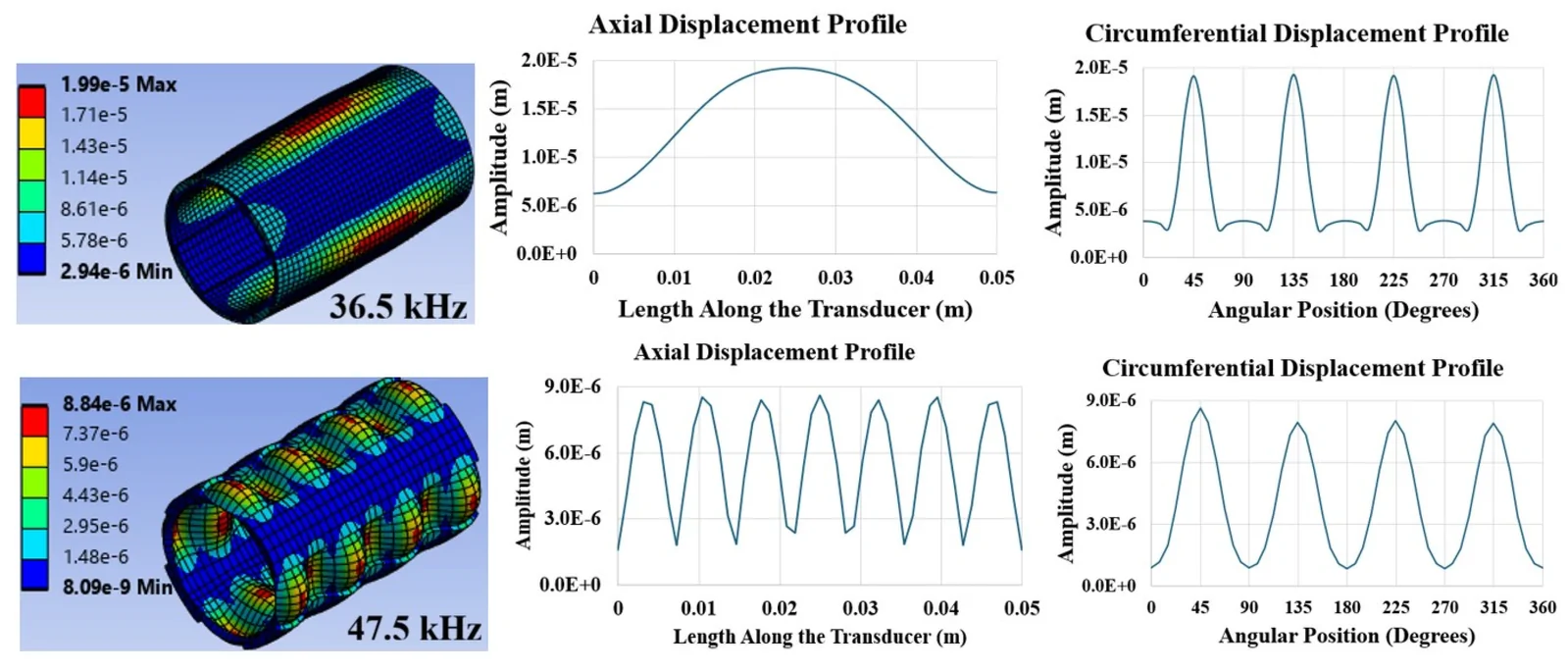
Mode shapes in 3D (deformations in meter), axial and circumferential wave profiles. (**Top**): Four internal circumferential steps at 36.5 kHz. (**Bottom**): Four external circumferential steps at 47.5 kHz.

**Figure 7:**
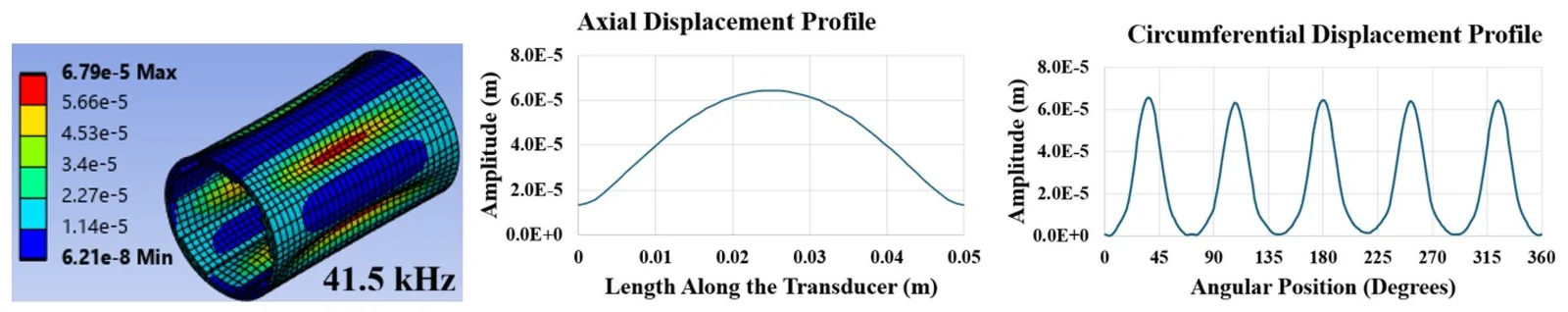

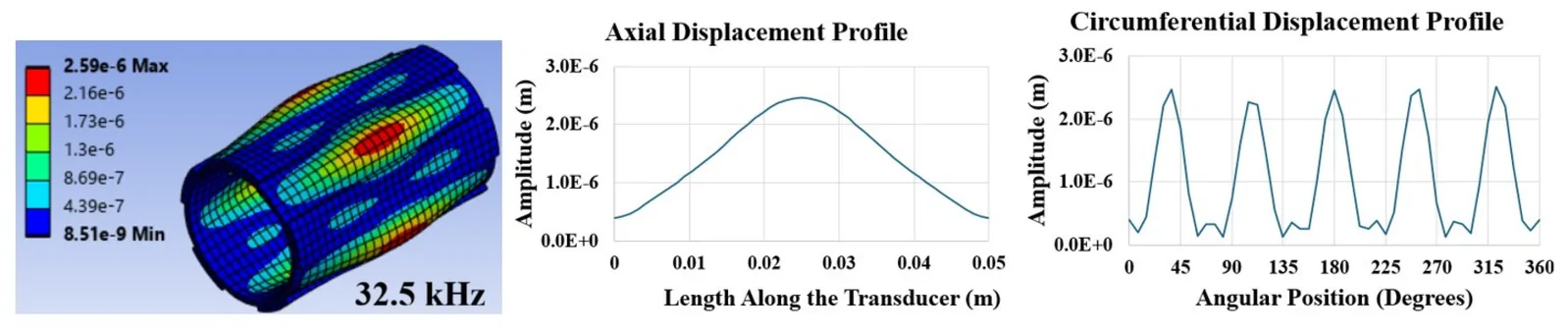
Mode shapes in 3D (deformations in meter), axial and circumferential wave profiles. (**Top**): Five internal circumferential steps at 41.5 kHz. (**Bottom**): Five external circumferential steps at 32.5 kHz.

**Figure 8:**
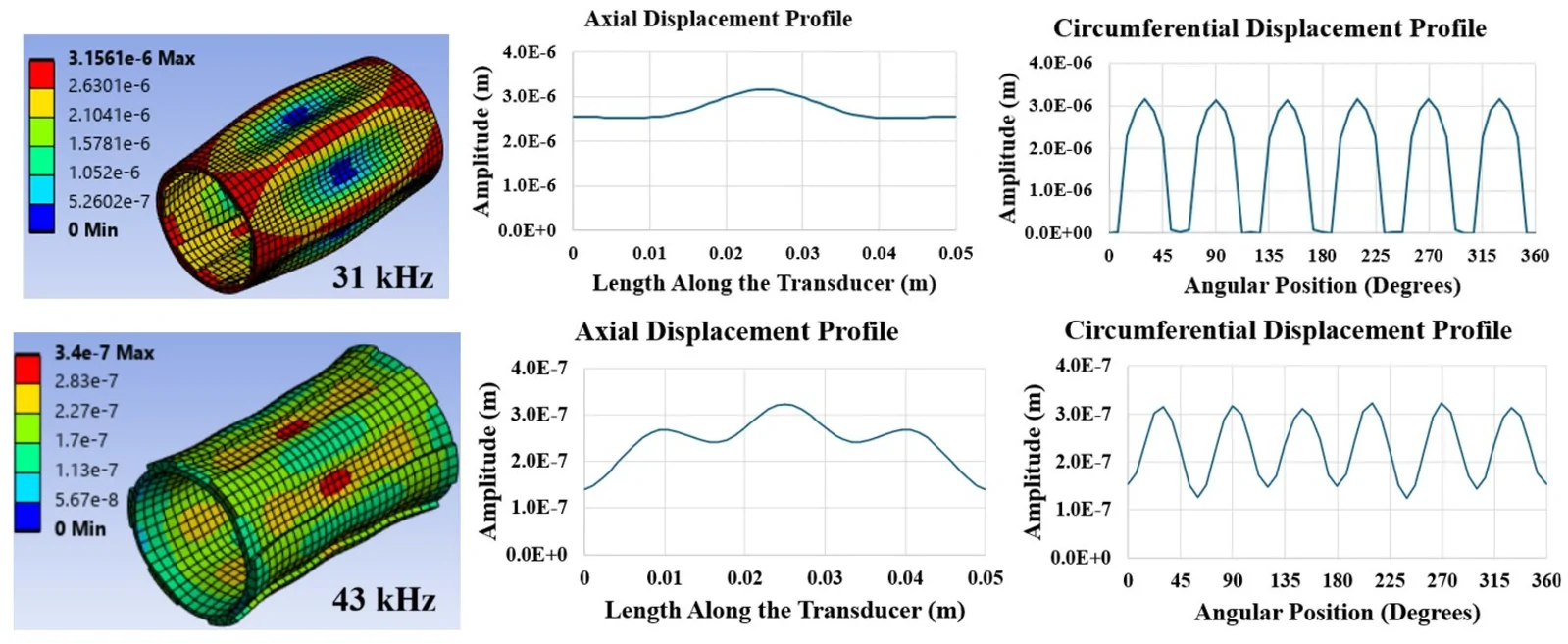
Mode shapes in 3D (deformations in meter), axial and circumferential wave profiles. (**Top**): Six internal circumferential steps at 31 kHz. (**Bottom**): Six external circumferential steps at 43 kHz.

**Figure 9:**
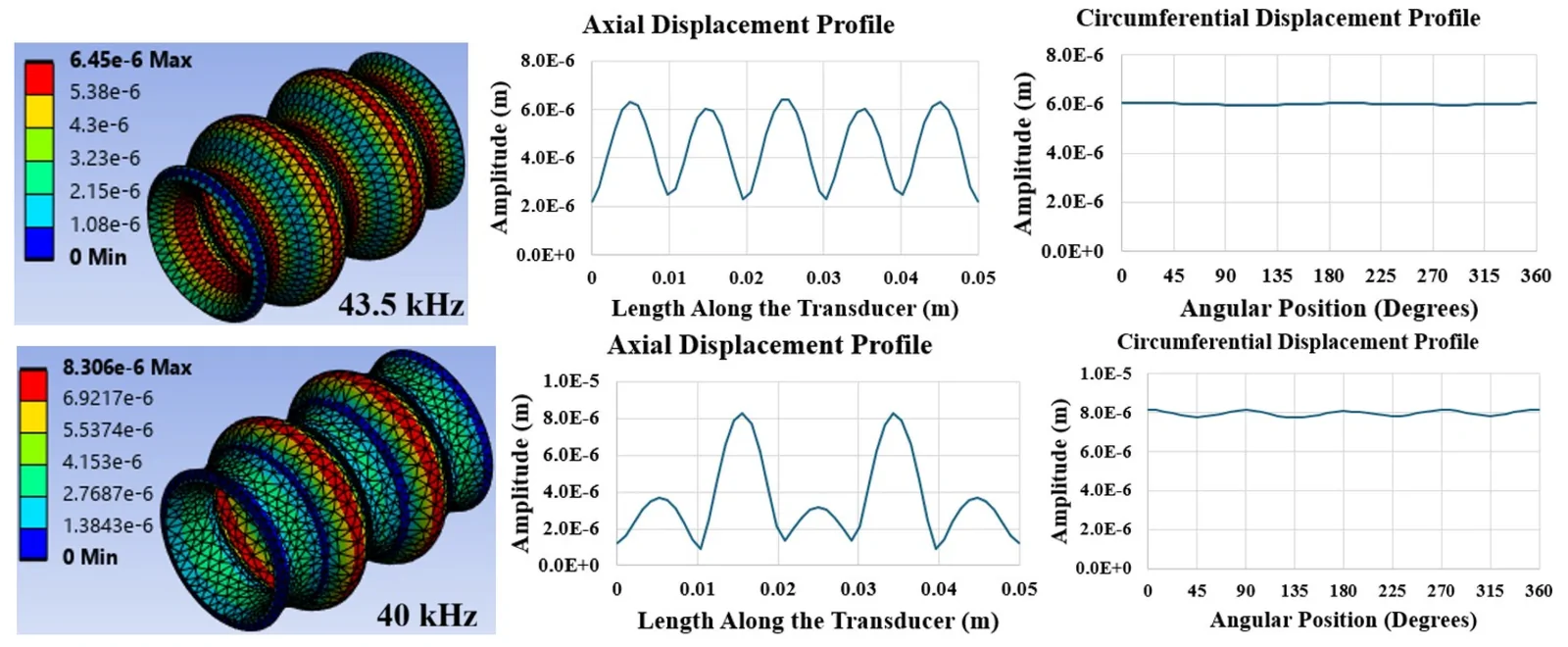
Mode shapes in 3D (deformations in meter), axial and circumferential wave profiles. (**Top**): Uniform thickness at 43.5 kHz. (**Bottom**): Two internal–external axial steps at 40 kHz.

Simulations were done and the results revealed that circumferential modes are absent within the frequency range of 30–50 kHz for the uniform thickness transducer. Subsequently, circumferentially stepped transducers were assessed for mode shapes with circumferential mode numbers potentially equal to the number of steps within the same frequency range of 30–50 kHz. The results are depicted in Figure 4, Figure 5, Figure 6, Figure 7, Figure 8 and Figure 9 illustrates the uniform thickness transducer as well as the two internal–external axially stepped transducers with axial modes. In all these figures, frequencies are given on each and deformations are in meter. For each case, axial and circumferential displacement profiles are provided illustrating the mode shape axial and circumferential wave numbers (m, n). For instance, in Figure 5, three external circumferentially stepped transducer has seven peaks in the axial displacement profile and three peaks in the circumferential displacement profile, corresponding to the mode shape (7, 3) whose 3D representation is depicted as well.

**Figure 10:**
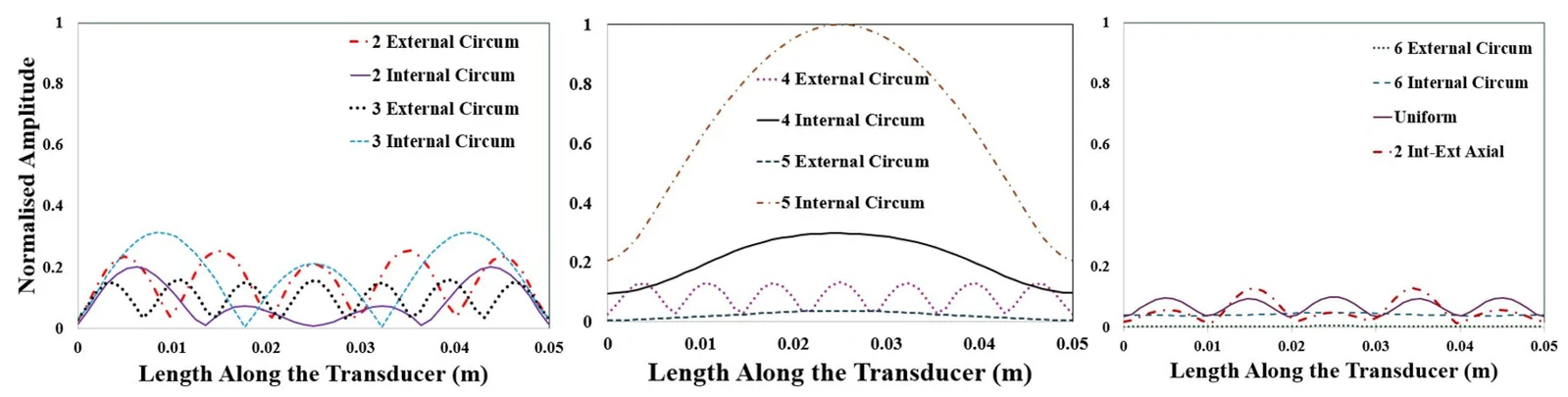
Normalized axial displacement profiles for all transducers (Int = internal, Ext = external, Circum = circumferential).

**Figure 11:**
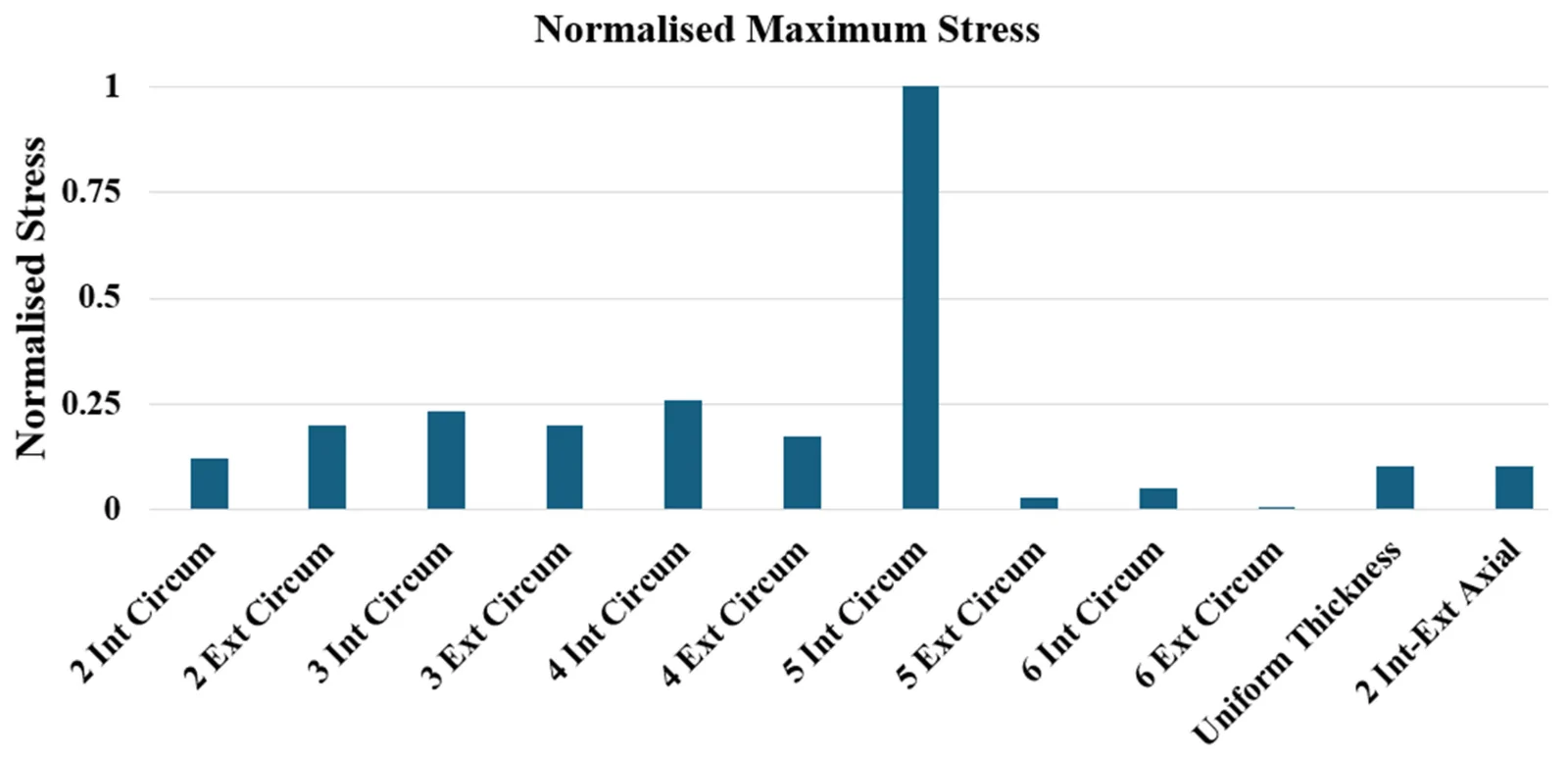
Normalized maximum stress for all transducers (Int = internal, Ext = external, Circum = circumferential).

As stated earlier, the amplitudes should not be treated as absolute values but should be considered in relative terms. Hence, Figure 10 illustrates the normalized amplitude for axial displacement profiles of all the transducers for the mode shapes in Figure 4, Figure 5, Figure 6, Figure 7, Figure 8 and Figure 9. Similarly, Figure 11 depicts the normalized maximum stress for each of these.

As evident from Figure 4, Figure 5, Figure 6, Figure 7 and Figure 8, all the circumferential displacement profiles have the same number of peaks as the number of steps with varying numbers of peaks in axial displacement profiles. This may suggest that the circumferential steps can effectively excite circumferential mode shapes which are absent for the uniform thickness transducer at the same frequency range of 30–50 kHz.

Figure 9 (top) for the uniform thickness transducer shows the mode shape with five equal peaks axially. In Figure 9 (bottom), the two internal–external axially stepped transducers have a pretty similar mode shape with two noticeable peaks in the axial displacement profile corresponding to two axial steps. There are also three small peaks which are at a much lower amplitude compared to the two main peaks and can be considered relatively stationary. The maximum amplitude of the stepped transducer is nearly 30% more than that of the uniform transducer. Hence, it may suggest that axial steps effectively localize and amplify the vibration amplitude within the stepped regions while making non-stepped regions relatively stationary.

Last but not least is the stress concentration at the sharp edges of the steps which can cause fatigue failure [23]. A noticeable observation from Figure 10 and Figure 11 is that the normalized amplitude and maximum stress for some transducers and particularly for the five internal circumferentially stepped transducers are much higher than those of the other transducers which are relatively within the same range and closer to each other. This is an important consideration for employing stepped transducers. One approach to minimize this high vibration and stress amplitude is to run the transducers at lower input excitation voltages which could result in lower vibration amplitudes and help with fatigue life [24]. Alternatively, the edges of the steps could be machined with a very small radius of curvature such as that seen in Figure 2c. One other approach could be making changes to the thickness of the transducer and/or the stepped region, and thickness variations. Hence, there are different strategies which could be employed to minimize stress concentration and enhance fatigue life. Deciding on one method may depend on the specific application, design restrictions and requirements. Therefore, proper design and analysis could help with this issue.

### 3.2. Experimental Results

**Figure 12:**
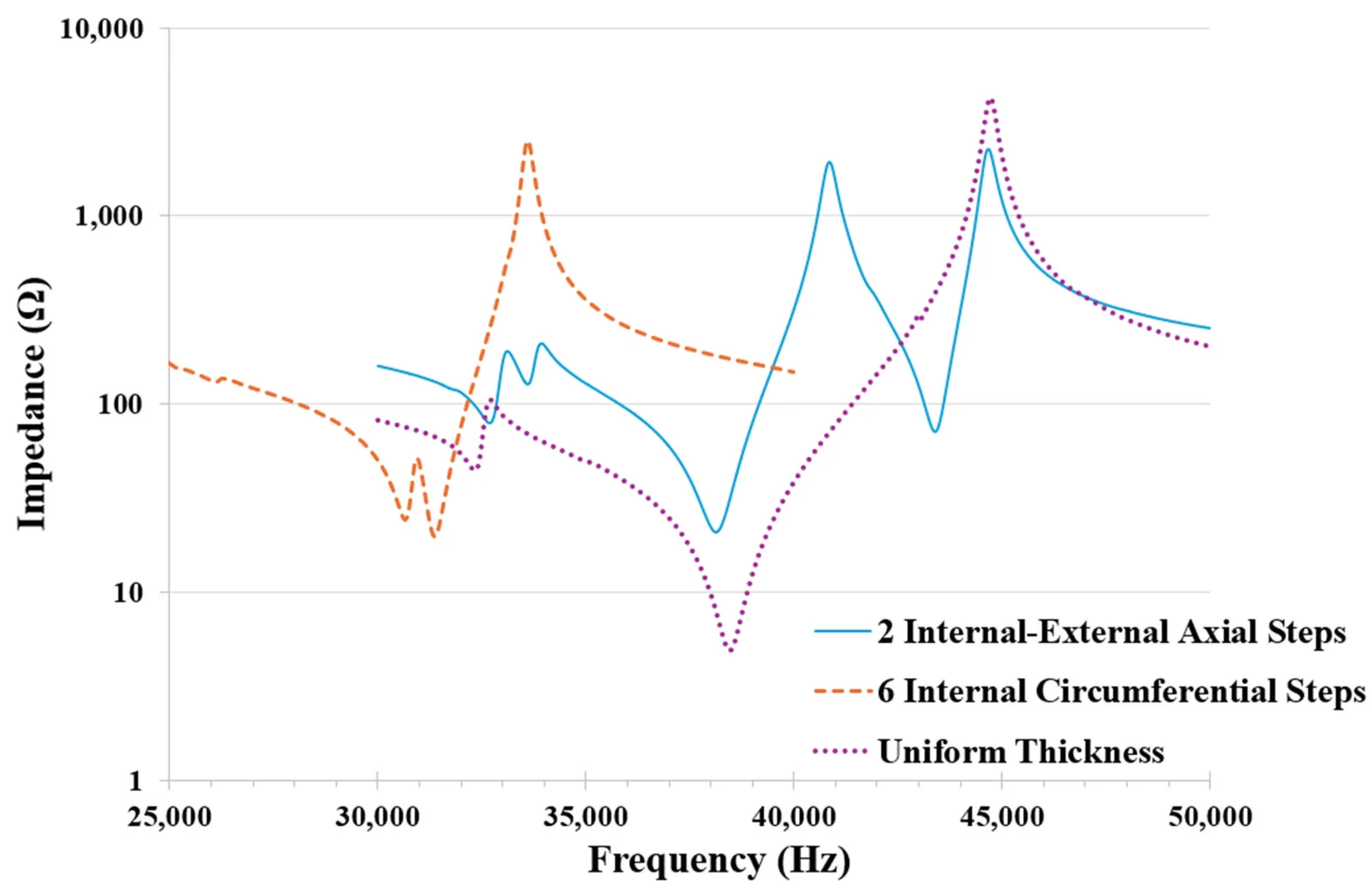
Impedance frequency response for experimentally tested transducers.

Experiments were conducted for the transducers shown in Figure 2 as per the experimental details discussed earlier. Impedance frequency response curves are presented in Figure 12. Table 3 shows the impedance analyzer results such as resonance and anti-resonance frequencies as well as the effective electromechanical coupling factor, $k_{eff}$, calculated using (Equation (8))

(8) $$k_{eff}=\sqrt{\frac{f_{AR}^{2}-f_{R}^{2}}{f_{AR}^{2}}}$$

where *f_AR_* and *f_R_* are anti-resonance and resonance frequencies, respectively corresponding to the maximum and minimum impedance. Table 3 also reveals the operating deflection shapes detected by the vibrometer FastScan mode, while the transducer is driven at different frequencies swept within the same 30–50 kHz range to obtain mode shapes of interest previously observed in simulations to validate them. These are represented in Table 3 using (m, n) being the axial and circumferential wave numbers, respectively.

The uniform thickness transducer mode shape of (5, 0) shown in Figure 9 at 43.5 kHz was experimentally observed at 43 kHz by the Laser Scanning Vibrometer which is a very good agreement. However, this mode was not observed by the impedance analyzer. This resonance is electrically suppressed in the impedance spectrum due to a combination of partial spatial charge cancelation, where alternating tension and compression zones neutralize generated charges, and capacitive masking, where the inactive uniform electrode geometry creates a large static capacitance that shunts the remaining weak motional signal.

The two internal–external axially stepped transducer mode shape of (5, 0) was experimentally observed at 43 kHz by the Laser Scanning Vibrometer compared to the 40 kHz in simulations which is a reasonable agreement. The impedance analyzer resonance was found to be at 43.38 kHz and anti-resonance of 44.64 kHz giving an effective electromechanical coupling factor of 0.24. Although this mode has five peaks similar to the uniform thickness transducer, three peaks are at a much lower amplitude and relatively stationary as discussed earlier and evident in Figure 9. Hence, the issue of not being detected by the impedance analyzer does not appear here.

The six internal circumferentially stepped transducer mode shape of (1, 6) was observed at 31 kHz both experimentally and in simulations. The impedance analyzer showed a resonance and anti-resonance of 31.37 kHz and 33.63 kHz, respectively, which gives an effective electromechanical coupling factor of 0.36.

Experimental validation of these mode shapes for the stepped transducers may suggest that stepped-thickness designs can help to control and localize vibration mode shapes for cylindrical piezoelectric transducers. This may introduce new avenues in their uptake and applications.

The impedance frequency response for the uniform thickness transducer is shown in Figure 12. With the resonance at 38.47 kHz and anti-resonance at 44.69 kHz, an effective electromechanical coupling factor of 0.51 is obtained, as in Table 4. This corresponds to the mode shape of (1, 0), breathing mode, which was experimentally identified at 37.4 kHz by the Laser Scanning Vibrometer and was found at 38 kHz in simulations. All simulation and experimental frequencies are in close agreement.

Figure 12 and the impedance frequency response for the two internal–external axially stepped transducer reveals another pair of resonance–anti-resonance at 38.12 kHz and 40.83 kHz. This yields an effective electromechanical coupling factor of 0.36, as in Table 4, and corresponds to the mode shape (1, 0), the breathing mode. This was experimentally observed at 37 kHz and at 36 kHz in simulations which show reasonable agreement.

According to the results in Table 3 and Table 4, the uniform thickness transducer has an effective electromechanical coupling factor of 0.51. Two internal–external axially stepped transducers exhibit lower effective electromechanical coupling factors of 0.24 and 0.36 at different resonant modes and frequencies. Similarly, the six internal circumferentially stepped transducer shows an effective electromechanical coupling factor of 0.36. These lower values indicate that stepped transducers are less efficient at converting energy between the electrical and mechanical domains, resulting in a narrower bandwidth. While this limits their high-power or wide-frequency applications, this may suggest that stepped designs may be suitable for applications requiring extreme precision, stability, or narrow-frequency selection. This may also enhance their uptake in such application areas.

### 3.3. Application Results

**Figure 13:**
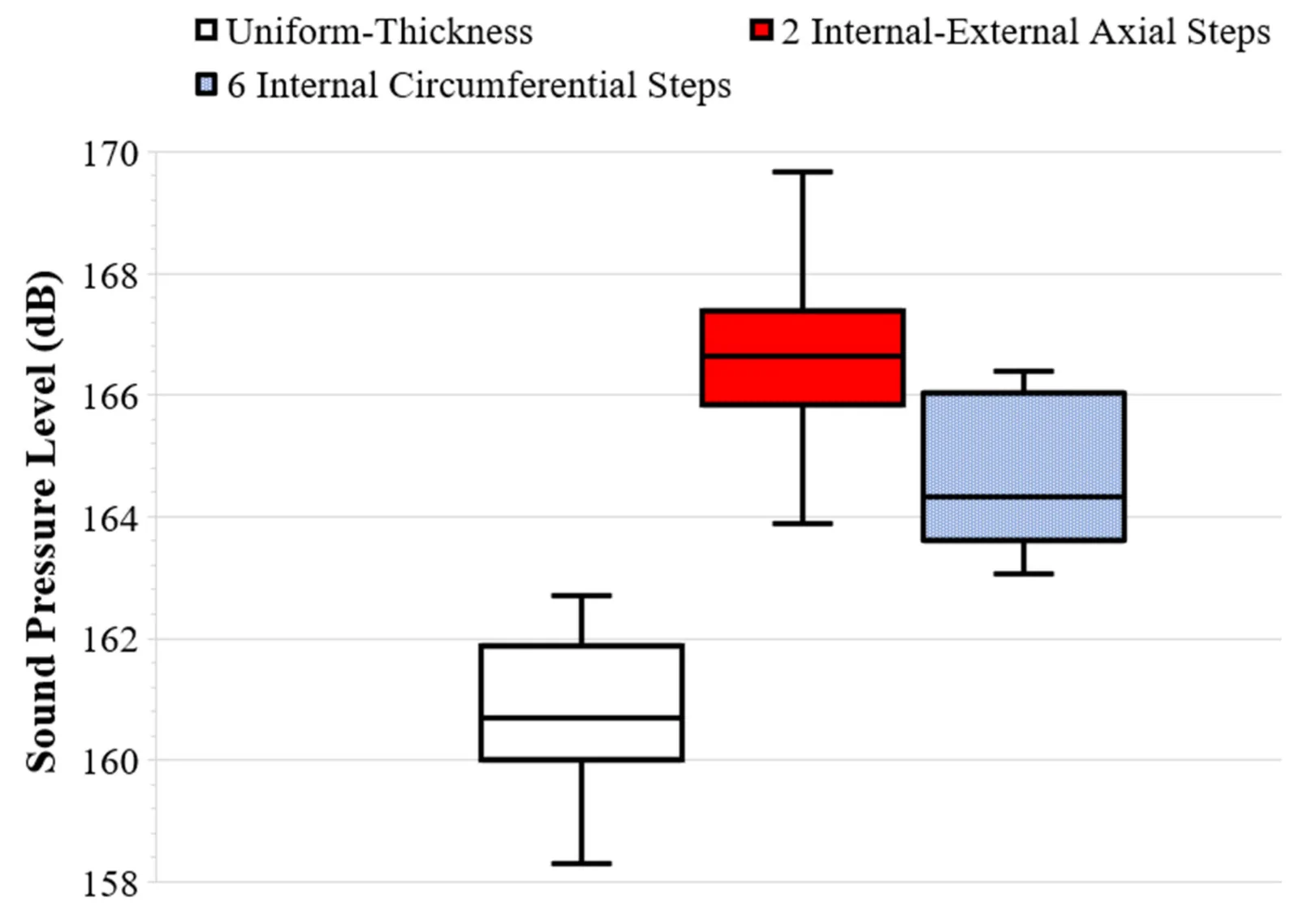
Box charts for statistical acoustic results.

In order to evaluate the performance of these stepped designs, some experiments were conducted on the application of them as an acoustic actuator. Uniform thickness and the two stepped transducers were driven within the same frequency range of 30–50 kHz and the generated acoustic field inside them was measured continuously using the microphone module to identify the maximum. Typical results are presented in Table 5 and box charts are provided in Figure 13 for statistical results. As evident from the Table and Figure, the two internal–external axially stepped transducers amplify the output acoustic pressure nearly two times whereas the six internal circumferentially stepped transducers amplify that by around 1.5 times. This may indicate that the stepped designs, leading to mode shape control and localization, can enhance the acoustic performance of the transducers. The maximum acoustic pressure for all three transducers occurred at pretty similar frequencies and mode shapes reported earlier in Table 3 (close to 43 kHz for both the uniform and two internal–external axially stepped, and close to 31 kHz for the six internal circumferentially stepped). As stated earlier, the two internal–external axially stepped transducers have two in-phase peaks of higher amplitude and the other three peaks are relatively stationary. However, for the uniform thickness transducer, the alternate peaks are in counter-phase leading to radiated waves canceling each other. Accordingly, the stepped transducer has amplified acoustic pressure. For the six internal circumferentially stepped transducers, the vibration localization with six in-phase peaks helps to amplify the acoustic pressure.

## 4. Conclusions

This study investigates piezoelectric cylindrical transducers using ANSYS simulations and experimental validations with the aim of controlling their vibration characteristics, mode shapes and amplitude. To this aim, stepped-thickness configurations with axial and circumferential steps were considered. Steps were introduced on the inner and outer surface of these transducers and simulations were performed to obtain their mode shapes and to investigate the effectiveness of these steps in controlling mode shapes, exciting certain mode shapes and affecting the amplitude. The results showed that circumferential steps effectively excite mode shapes with matching circumferential mode numbers. For axial steps, it was observed that the steps can further localize and amplify the vibration amplitude of the axial modes compared to the uniform thickness transducer. Subsequently, to validate these observations in simulations, axially and circumferentially stepped transducers were experimentally driven as well as the uniform thickness transducer. Their vibration was captured using Laser Scanning Vibrometer providing the same mode shapes as in simulations at very close frequencies. The impedance frequency response of the transducers was also obtained indicating that steps reduce the effective electromechanical coupling factor. However, they can provide a narrowband transducer suitable for many precision applications. Further, some application tests were conducted to evaluate the performance of transducers as acoustic actuators. The results showed enhanced and amplified acoustic pressures with the stepped transducers. These may indicate the efficacy of these steps in vibration control of cylindrical piezoelectric transducers and aim at enhancing their uptake for various industrial applications.

## Figures and Tables

**Table 1:** Material properties [27].

Material	Density (kg/m^3^)	Elastic Stiffness Matrix Components (Pa)					Relative Permittivity		Stress Constant (C/m^2^)		
		${c_{11}}^{E}$	${c_{12}}^{E}$	${c_{13}}^{E}$	${c_{33}}^{E}$	${c_{44}}^{E}$	ε11sε0	ε33sε0	$e_{31}$	$e_{33}$	$e_{15}$
PZT-5L	7600	1.21 × 10^11^	7.54 × 10^10^	7.52 × 10^10^	1.11 × 10^11^	2.11 × 10^10^	916	830	−5.4	15.8	12.3

**Table 2:** Element and mesh details (Circum = circumferential, Int = internal, Ext = external).

		2 Circum		3 Circum		4 Circum		5 Circum		6 Circum		2 Axial	Uniform Thickness
		Int	Ext	Int	Ext	Int	Ext	Int	Ext	Int	Ext	Int-Ext	
Skewness	Average	0.31	0.31	0.36	0.31	0.39	0.24	0.35	0.31	0.34	0.39	0.44	0.29
	Max	0.65	0.57	0.73	0.63	0.62	0.63	0.78	0.75	0.67	0.71	0.85	0.75
Element	Type	SOLID226										SOLID227	
	Number	2015	2048	1620	2244	3936	2139	2294	1820	2542	1876	12,310	9800
	Size × $10^{-3}$	2.6	2.5	2	2.5	2	2.8	3	2	2.4	2	2.3	1.8

**Table 3:** Experimental results compared to relevant simulation results.

Transducer	Mode Shape (m, n)	FEA (ANSYS)	Experimental		Effective Electromechanical Coupling Factor *k_eff_*
		Frequency (kHz)	Laser Scanning Vibrometer Frequency (kHz)	Impedance Analyzer Resonant Frequency (kHz)	
Uniform Thickness	(5, 0)	43.5	43	- *	- *
2 Internal–External Axial	(5, 0)	40	43	43.38 ^^^	0.24
6 Internal Circumferential	(1, 6)	31	31	31.37 ^#^	0.36

* This mode and frequency were not identified by the Impedance Analyzer. ^^^ Anti-resonance of 44.64 kHz. ^#^ Anti-resonance of 33.63 kHz.

**Table 4:** Experimental results compared to relevant simulation results for other vibration modes of the uniform thickness and 2 internal–external stepped transducers.

Transducer	Mode Shape (m, n)	FEA (ANSYS)	Experimental		Effective Electromechanical Coupling Factor *k_eff_*
		Frequency (kHz)	Laser Scanning Vibrometer Frequency (kHz)	Impedance Analyzer Resonant Frequency (kHz)	
Uniform Thickness	(1, 0)	38	37.4	38.47 *	0.51
2 Internal–External Axial	(1, 0)	36	37	38.12 ^#^	0.36

* Anti-resonance of 44.69 kHz. ^#^ Anti-resonance of 40.83 kHz.

**Table 5:** Acoustic results for experimentally tested transducers.

Transducer	Frequency (kHz)	Microphone		
		Max Output Voltage (mV)	Max Output SPL (dB)	Corresponding Output Pressure (Pa)
Uniform Thickness	43.10	1080	160.69	2165.36
2 Internal–External Axial Steps	42.87	2180	166.79	4370.49
6 Internal Circumferential Steps	31.5	1700	164.63	3408.24

## Data Availability

Data available upon request from the corresponding author.
